# Association Between Asthmatic Patients’ Asthma Control Test Score and the Number of Exacerbations per Year in King Abdulaziz Medical City, Riyadh

**DOI:** 10.7759/cureus.24001

**Published:** 2022-04-10

**Authors:** Majed Alghamdi, Ziad A Aljaafri, Khalid H Alhadlaq, Sultan A Alamro, Saud M Alfaryan, Osama Al Swaidan, Mohamud Mohamud

**Affiliations:** 1 Pulmonary Medicine, Ministry of the National Guard Health Affairs, Riyadh, SAU; 2 College of Medicine, King Saud bin Abdulaziz University for Health Sciences, Riyadh, SAU; 3 Medical Education, King Saud bin Abdulaziz University for Health Sciences, Riyadh, SAU

**Keywords:** respiratory disease, wheezing, asthma exacerbations, act, asthma

## Abstract

Background

Asthma is a reactive airway disease that has a high prevalence across the globe. Asthma exacerbations can occur due to various bacterial and viral infections that irritate nerve endings in the airways. With time, airway obstruction follows, and patients with asthma have various symptoms that occur intermittently. Asthma symptoms primarily include breathlessness, wheezing, coughing, and chest tightness. This research focused on the association between the Asthma Control Test (ACT) score and number of exacerbations per year.

Methods

A questionnaire-based, cross-sectional study was conducted at the outpatient pulmonary clinic, King Abdulaziz Medical City, a tertiary hospital in Riyadh. The study included 227 adult patients who were diagnosed with asthma and had no other pulmonary diseases or other medical diseases that could mimic asthma exacerbation. Data was collected by direct interview with the patients and through the BESTCare system in King Abdulaziz Medical City. All the data were collected through Microsoft Excel 2010 (Microsoft, Redmond, WA, USA) and analyzed using Statistical Package for Social Sciences (SPSS) Statistics version 23 (IBM Corp., Armonk, NY, US). The categorical data we used were presented by percentages and frequencies such as gender, whereas the numerical data were prescribed as mean and standard deviation such as age and number of exacerbations. For inferential statistics, Chi square was used to find the association between the categorical variable while T-test and ANOVA test were used to find the relationship between asthma control test score of asthmatic patients, which was divided into three different groups based on their scores that include: well-controlled, partially controlled, or uncontrolled, and the number of exacerbations per year.

Results

A total of 227 adult asthma patients were enrolled in this study, most of them were females (72.7%). Average age of the participants was 47.3 ± 13.8 years. The average ACT score was found to be 18.5 ± 4.9 out of 25. Uncontrolled asthma was present in 26% of the patients, 22.9% were partially controlled and 51.1% had well-controlled asthma; to relieve the exacerbation most of the patients used salbutamol (51.5%), 35.2% used oxygen and 30.4% did not use any medication. Gender and age were not associated with ACT score (P = 0.787 and 0.797, respectively), whereas number of exacerbations was significantly associated with ACT score (P = 0.000), as fewer exacerbations were reported with higher ACT scores.

Conclusion

About one-quarter of the patients had uncontrolled asthma, slightly less than one-quarter of the patients had partially controlled asthma while more than half of the patients had well-controlled asthma. Number of exacerbations was found to be significantly associated with asthma control test score as fewer exacerbations were reported in well-controlled asthmatic patients.

## Introduction

Asthma is a disease of airway hyperreactivity and bronchospasm. Asthma exacerbations can occur due to various bacterial and viral infections that further irritate nerve endings in the airways [1]. With time, airway obstruction follows, due to the severe inflammation and bronchospasm, muscle constriction surrounding the airways. Patients with asthma have various symptoms that occur intermittently. Asthma symptoms primarily include breathlessness, wheezing, coughing, and chest tightness. Usually, asthma patients experience acute or subacute exacerbations in symptoms that worsen over time [2]. Throughout an asthma exacerbation, the airways become swollen and inflamed. In addition, the muscles around the airways contract and the airways produce extra mucus, causing remodeling of the airways and bronchial hyperresponsiveness. During an attack, the patient may experience coughing and wheezing in addition to dyspnea. Symptoms of a minor asthma attack can be controlled with rapid home treatment. However, severe asthma attacks that don't respond to home treatment can be a serious medical emergency. The best way to prevent an asthma exacerbation is by identifying and treating the triggering factors as soon as possible [3]. Many factors have been identified regarding their association with asthma attacks. Asthma is more predominant and serious among young boys; however, there is a switch between genders at puberty, which has been linked to the rise of sex hormones [4]. Moreover, the progress from youth to adulthood is characterized by a higher chance ratio of persistence of wheezing in females, and by improvement in males but worsening in females [4]. A potential clarification could have to do with the association between asthma attacks and the menses, which is a perceived variable of asthma worsening [5]. An asthmatic patient can live a fully active life by following a strict management plan provided by a physician, but there is no definite cure for the disease. Management usually involves being aware of the patient’s triggers, then taking steps to avoid them. In order to prevent possible exacerbations of symptoms, continuous control and avoiding triggers that may cause asthma exacerbations are essential [6]. Short-term medications such as ipratropium, short-acting beta-agonists, and intravenous or oral corticosteroids are used to relieve the symptoms. People who suffer from persistent symptoms must take long-term medications including leukotriene modifiers, inhaled corticosteroids, theophylline, and long-acting beta-agonists [7].

Asthma is considered one of the most identified chronic diseases in Saudi Arabia. The prevalence of asthmatics in Saudi Arabia is 11.3%, while it is about 4.3% of the worldwide population, and both numbers are rising annually [8]. Average asthma prevalence annually is higher in children than in adults [9]. Based on studies conducted over the past three decades, the annual increase in prevalence may be attributed to sudden changes in dietary habits, lifestyle, and exposure to environmental factors, such as sandstorms, dust, indoor allergens, and tobacco [10]. The prevalence of asthma also increases with every successive lower poverty level group [9].

The Asthma Control Test (ACT) is used to measure how well-controlled a patient’s asthma is. Asthmatics with higher ACT scores predict that the likelihood of asthma exacerbations is lower. The plan was to understand how effective the ACT score is in predicting the outcome of patients to optimize its benefits. Moreover, there was a lack of studies regarding the association of emergency department visits related to asthma exacerbations. Therefore, we thought of its importance as well as looked forward to understanding the relationship between ACT and the number of exacerbations.

Based on the ACT of asthmatic patients, we aimed to have a clear vision of controlled and uncontrolled asthmatic patients and to understand the association between asthmatic patients’ ACT and the possible number of exacerbations per year. The aim of the study was to determine the association between the ACT score and the number of exacerbations per year at King Abdulaziz Medical City (KAMC) in Riyadh.

## Materials and methods

A questionnaire-based, cross-sectional study was conducted at the outpatient pulmonary clinic, King Abdulaziz Medical City, a tertiary hospital in Riyadh. The sample size was designed to ensure 95% confidence interval, with a margin of error of 5%. The number of asthmatic patients in Saudi Arabia from the ages of 15 to 60 years old is estimated to be 530,000 patients as of 2016 [11]. The prevalence of exacerbations per year is approximately 18.2% [8] of total asthmatics (96,500 asthmatic patients). Using the Raosoft sample size calculator, the sample size of patients for this study was calculated. The study included 227 adult patients who were diagnosed with asthma and had no other pulmonary diseases such as chronic obstructive pulmonary disease, interstitial lung diseases, obstructive sleep apnea, or lung cancer. In addition to the absence of other medical diseases that could mimic asthma exacerbation such as systolic heart failure, end stage renal failure, or liver failure. Moreover, the sampling technique that has been used was a convenient sampling technique for eligible patients who came to the clinic.

Data was collected by direct interview with the patients and through the BESTCare system in KAMC. The first step was to use the ACT to detect the score for each patient that visited the pulmonary clinic, and the second step regarding the number of exacerbations we used a data collection sheet that was prepared by the research team based on the abstraction of data from the BESTCare system. The Arabic version of the asthma control test is valid, reliable, and has been used in the assessment of asthma since its validation [12]. It has been used to interview Arabic-speaking patients. Moreover, the English version was used too. The ACT is a patient self-administered tool to assess how well the asthma is controlled, composed of five questions with a maximum score of 25. Regarding the scoring, the ACT was divided into three different categories: 1. Well-controlled >= 20, 2. Partial control 16-19, 3. Uncontrolled <16. Furthermore, the collection sheet that was developed by the research team included the number of emergency department visits by the patients.

All the data were collected through Microsoft Excel (Microsoft, Redmond, WA, USA) and transferred to Statistical Package for Social Sciences (SPSS) version 23 (IBM Corp., Armonk, NY, USA) for analysis. Data were checked for any missing information and new variables were recorded and computed based on the data extracted. The categorical data we used were presented by percentages and frequencies such as gender, whereas the numerical data were prescribed as mean and standard deviation such as age and number of exacerbations. For inferential statistics, Chi square was used to find the association between the categorical variable while T-test and ANOVA were used to find the relationship between asthma control test score of asthmatic patients, which was divided into three different groups based on their scores that include: well-controlled, partially controlled, or uncontrolled, and the number of exacerbations per year. Regarding ethical consideration, all patients were given an informed consent before the ACT was conducted, and King Abdullah International Medical Research Center (KAIMRC) issued approval SP20/342/R.

## Results

Demographic and clinical characteristics of the study participants

A total of 227 adult patients who were diagnosed with asthma were included in this study. Most of them were females (72.7%). The average age of the participants was 47.3 ± 13.8 years (range from 18 to 68). Most of the participating patients (81.5%) had no visits to KAMC Emergency Department and 38 (16.7%) had less than five visits.

**Figure 1:**
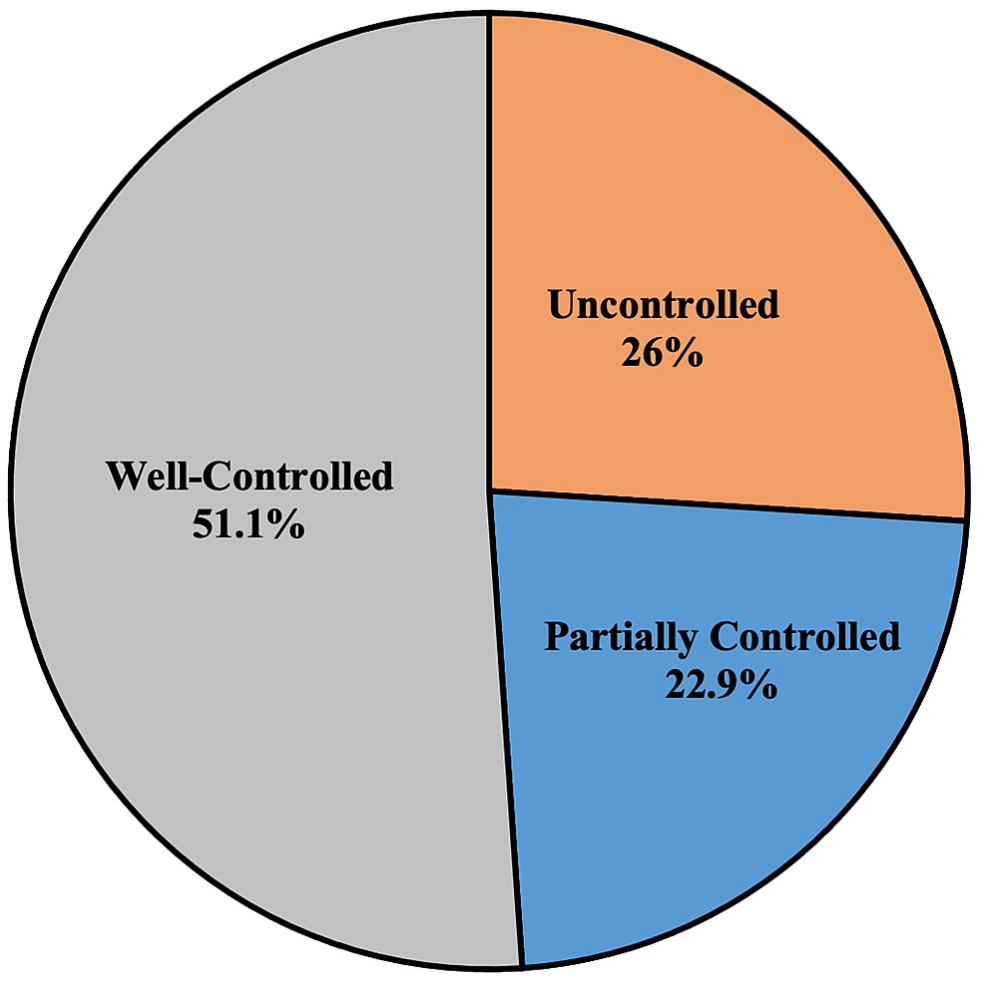
Frequency of Asthma Control Test categories among the study population

The average ACT score among the study population was found to be 18.5 ± 4.9 out of 25. Uncontrolled asthma was present in 26% of patients, partially controlled asthma was present in 22.9% while the majority of patients (51.1%) had well-controlled asthma (Figure 1). Table 1 presents a summary of the results for the demographic and clinical characteristics of the study population. 

**Table 1:** Demographic & clinical characteristics of the participants (n=227) ACT: Asthma Control Test; KAMC: King Abdulaziz Medical City

Variable	Category	Frequency	Percent
Age (years)	Mean ± SD	47.3 ± 13.8 (range from 18 to 68)	
Gender	Male	62	27.3%
	Female	165	72.7%
Number of KAMC Emergency Department visits	No visit	185	81.5%
	< 5	38	16.7%
	≥ 5	4	1.8%
ACT Score	Mean + SD	18.5 ± 4.9 (range from 6 to 25)	
Emergency Department visit	Yes	114	50.2%
	No	113	49.8%
Number of exacerbations	0	73	32.2%
	1-4	110	48.5%
	5-10	44	19.4%

About the agents used, the most used one was salbutamol (62.6%) followed by Symbicort (44.1%), Seretide (39.6%), montelukast (37.4%) and mometasone (15%) (Table 2). Concerning the agents used to relieve the exacerbation, nearly half of the patients used salbutamol, about one-third used oxygen and slightly less than one-third did not use any medication.

**Table 2:** Agents used * Agents used for acute exacerbation management.

Agent	Frequency	Percent
Salbutamol*	142	62.6%
Prednisone*	5	2.2%
Prednisolone*	4	1.8%
Tiotropium bromide*	25	11%
Seretide (Fluticasone propionate / Salmeterol)	90	39.6%
Montelukast	85	37.4%
Symbicort (Budesonide / Formoterol)	100	44.1%
Mepolizumab	2	0.9%
Loratadine	1	0.4%
Omalizumab	3	1.3%
Budesonide	7	3.1%
Fluticasone	18	7.9%
Mometasone	34	15%
Dupilumab	1	0.4%
Vilanterol inhaler	6	2.6%

Association between ACT score and gender, age, and number of exacerbations per year

The results of the analysis revealed that gender and age were not associated with ACT score, as the calculated P values were recorded as 0.787 and 0.797, respectively, whereas number of exacerbations last year was associated with ACT score (P = 0.000). Our results showed that lower number of exacerbations (0 to four) was predominant among well-controlled patients while higher number of exacerbations (five to 10) was predominant among uncontrolled patients (Table 3).

**Table 3:** Association between asthmatic patients’ Asthma Control Test (ACT) score and gender, age and the number of exacerbations per year * Chi square test, ** One way ANOVA test.

Variable	ACT Score			P value
	Uncontrolled	Partially Controlled	Well-Controlled	
	N (%)			
Gender				
Male	15 (24.2%)	13 (21%)	34 (54.8%)	0.787*
Female	44 (26.7%)	39 (23.6%)	82 (49.7%)	
Number of exacerbations				
0	1 (1.4%)	16 (21.9%)	56 (76.7%)	0.000*
1-4	30 (27.3%)	26 (23.6%)	54 (49.1%)	
5-10	28 (63.6%)	10 (22.7%)	6 (13.6%)	
	Uncontrolled	Partially Controlled	Well Controlled	
	Mean (SD)			
Age	46.3 (12.8)	47.3 (15.5)	47.8 (13.7)	0.797**

Association between gender and number of exacerbations

The results of the analysis showed that there was no significant association between gender and number of exacerbations, as the calculated P value was recorded as 0.620 (Table 4).

**Table 4:** Association between gender and the number of exacerbations * Chi square test

Variable	Gender		P value
	Male	Female	
Number of exacerbations			
0	23 (37.1%)	50 (30.3%)	0.620*
1-4	28 (45.2%)	82 (49.7%)	
5-10	11 (17.7%)	33 (20%)	

## Discussion

Control of asthma is one of the most important determinants of progression of the disease and it was proven to be a critical factor affecting the frequency and severity of exacerbations. The aim of this study was to study and demonstrate the association between the patient's asthma control test score and the number of exacerbations per year.

In the current study more than two-thirds of the respondents were females, and the remaining were males, and this could be attributed to the relatively higher prevalence of asthma among females that has already been reported in many international registries as reported in other study showing prevalence of asthma among females is nearly two times its prevalence among males [13]. The average age of the respondents was 47 years old. Most patients had no visits to KAMC Emergency Department (81.5%).

Regarding the average ACT score among the study population, it was found to be 18.5 ± 4.9 out of 25. About one-quarter of the patients had uncontrolled asthma, slightly less than one-quarter of the patients had partially controlled asthma while more than half of the patients had well-controlled asthma, and this was found to be contradictory to another study which was conducted in Saudi Arabia showing that more than half of the patients had uncontrolled asthma [14]. 

About half of the patients had a previous visit or needed to go to the emergency department, this was reported in another parallel study which was conducted in the UK but with a lesser percentage of patients compared to the current study and this study showed only one-third of the patients needed emergency visit [15].

Regarding the number of exacerbations in the last year, nearly one-third of the patients had no exacerbation last year. Concerning the agent used to relieve the exacerbation; nearly half of the patients used salbutamol, about one-third used oxygen and slightly less than one-third did not use any medication. Similar results were found in another study which was conducted in the USA showing the most commonly used drug in treatment of acute exacerbations of asthma was found to be nebulized beta-2 agonist as salbutamol [16].

Concerning the most common drug used, the most used one was salbutamol which was reported by about two-thirds of the patients followed by Symbicort which was stated by nearly half of the patients, then Seretide, montelukast and mometasone each reported by more than one-third of the patients, controller therapy is most effective if administered as combination therapy rather than monotherapy as demonstrated in another study conducted in the Netherlands by Loymans et al. showing a combination of long-acting beta-2 agonist (LABA) and inhaled corticosteroids (ICS) is more effective and secure in preventing asthma exacerbations [17].

Regarding association of gender and age with ACT, gender and age were not found to be associated with ACT score, also the results of the analysis showed that there was no significant association between gender and number of exacerbations, and this was in contradiction to a parallel study conducted in Saudi Arabia showing association of gender with ACT with females having uncontrolled asthma more than males [18].

The number of exacerbations last year was found to be significantly associated with ACT score and our results showed that a lower number of exacerbations (0 to four) was predominant among well-controlled patients while a higher number of exacerbations (five to 10) was predominant among uncontrolled patients. The same results were found in another study conducted by van Dijk et al. in the Netherlands showing decreased number of exacerbations with increased asthma control score [19].

The limitations of the current article were the reality that information was just gathered from one center, potentially restricting the generalizability of the discoveries. This subject requires more exploration to be done in the area, with a bigger sample size and multiple centers involved to achieve an accurate estimation of how well asthma is controlled and what possible influencing factors causing exacerbations at the local and international levels are in order to achieve better control.

## Conclusions

About one-quarter of the patients had uncontrolled asthma, slightly less than one-quarter of the patients had partially controlled asthma while more than half of the patients had well-controlled asthma. Half of the patients had a previous visit or went to the emergency department. Nearly one-third of the patients had no exacerbation last year nearly half of them had one to four exacerbations. The most common drug used to relieve acute asthmatic exacerbations was salbutamol. Gender and age were not found to be associated with ACT score. Number of exacerbations last year was found to be significantly associated with ACT score as a lesser number of exacerbations was reported in well-controlled asthmatic patients.
